# Traditional Chinese Medicine Targeting Heat Shock Proteins as Therapeutic Strategy for Heart Failure

**DOI:** 10.3389/fphar.2021.814243

**Published:** 2022-01-18

**Authors:** Yanchun Wang, Junxuan Wu, Dawei Wang, Rongyuan Yang, Qing Liu

**Affiliations:** ^1^ Shenyang the Tenth People’s Hospital, Shenyang, China; ^2^ Shunde Hospital of Guangzhou University of Chinese Medicine, Foshan, China; ^3^ The Second Clinical School of Medicine, Guangzhou University of Chinese Medicine, Guangdong Provincial Hospital of Chinese Medicine-Zhuhai Hospital, Zhuhai, China

**Keywords:** heat shock proteins, heart failure, traditional Chinese medicine, myocardial injury, therapeutic targets

## Abstract

Heart failure (HF) is the terminal stage of multifarious heart diseases and is responsible for high hospitalization rates and mortality. Pathophysiological mechanisms of HF include cardiac hypertrophy, remodeling and fibrosis resulting from cell death, inflammation and oxidative stress. Heat shock proteins (HSPs) can ameliorate folding of proteins, maintain protein structure and stability upon stress, protect the heart from cardiac dysfunction and ameliorate apoptosis. Traditional Chinese medicine (TCM) regulates expression of HSPs and has beneficial therapeutic effect in HF. In this review, we summarized the function of HSPs in HF and the role of TCM in regulating expression of HSPs. Studying the regulation of HSPs by TCM will provide novel ideas for the study of the mechanism and treatment of HF.

## Introduction

Heart failure (HF) is a clinical syndrome that is characterized by impaired myocardial structure or ventricular contraction/diastolic function and it causes insufficient cardiac output (Yancy et al., 20132013). HF is a critical health problem that affects 26 million people worldwide, and an estimated 17–45% of patients with HF admitted to hospital die within 1 year of admission. Most patients die within 5 years after admission (Ambrosy et al., 2014; Ponikowski et al., 2014). The recommended pharmacological treatments for HF include angiotensin-converting enzyme inhibitors (ACEI), angiotensin receptor blockers (ARB), angiotensin receptor neprilysin inhibitor (ARNI), I_f_ channel inhibitor, β-adrenergic blockers and diuretics. Recommended treatments are able to reduce hospitalizations, morbidity and mortality, but can have severe side effects like angioedema, electrolyte depletion and fluid depletion (Yancy et al., 20172017). Therefore, developing new therapeutic methods and medicine will be of great significance in the treatment of HF.

Heat shock proteins (HSPs) are a group of conserved proteins with multiple biological activities (Stetler et al., 2010). Previous studies reveal the vital role played by HSPs in HF (Ranek et al., 2018). Therefore, it would be imperative to focus on regulation of HSPs in the treatment of HF. Traditional Chinese Medicine (TCM) contains numerous chemical components and active ingredients, which can regulate expression of HSPs in various diseases (Yang et al., 2017a; Kunde et al., 2017; Zhou et al., 2018; Zhao et al., 2020). Furthermore, TCM can improve cardiac function and ameliorate damage caused by HF (Wang et al., 2017a). Recent studies suggest that TCM can alter expression of HSPs in HF (Wang et al., 2014; Zhang et al., 2018a; Nie et al., 20192019). Consequently, TCM may regulate expression of HSPs to treat HF. We therefore summarized the role of HSPs in the pathogenesis of HF, effects of TCM in regulating HSPs and action of TCM targeting HSPs in treating HF to enhance our understanding of the mechanisms in HF, and provide novel ideas for its application as a therapeutic strategy of HF.

## HSPs Family

HSPs widely occur in eukaryotic cells and can respond to multiple stimuli, high temperature, lack of nutrients, energy depletion, aging, oxidative stress, acute and chronic inflammatory reactions, viral and bacterial infections, ischemia, heavy metals and excessive exercise (Kregel, 19852002). HSPs have a variety of biological functions. The most crucial role is they act as molecular chaperones which ensure correct folding of newly synthesized proteins, facilitating refolding of misfolded proteins upon stress, and maintaining protein structure and stability (Stetler et al., 2010). HSPs is divided into the following six families according to their relative molecular masses; HSP110, HSP90, HSP70, HSP60, HSP40, and small HSPs (HSPs).

HSP110 is a high molecular weight HSP belonging to HSP70 superfamily. Its expression is also induced by stress, and it cooperates with other HSPs to facilitate refolding of proteins and cell survival (Zuo et al., 2016).

HSP90 is a highly conserved ATP-dependent molecular chaperone that is involved in homeostasis and folding of proteins (Wu et al., 2017). The HSP90 family has two isoforms which occur in the cytoplasm: 1) stress-inducible HSP90α and 2) a constitutively expressed HSP90β.

HSP70 family is by far the most widely studied group of HSPs which generally occur in the cytoplasm and nucleus (Shrestha and Young, 2016). HSP70 acts in an ATP-dependent manner, and its family includes inducible HSP70, constitutively expressed HSP70 and glucose-regulated protein 78 (GRP78). The chaperone protein, HSP70 is principally dedicated to the degradation of unstable and misfolded proteins and refolding of proteins, preventing and dissolving protein complexes, and stabilizing cellular homeostasis (Daugaard et al., 2007). GRP78 belongs to the HSP70 family and plays an essential role in attenuating endoplasmic reticulum (ER) stress. ER is a cellular organelle responsible for storage of calcium, protein synthesis and folding, and lipid metabolism (Schwarz and Blower, 2016). Ischemia, hypoxia, disruption of calcium homeostasis, ATP depletion, and oxidative stress result in accumulation of unfolded proteins in the ER subsequently causing endoplasmic reticulum (ER) stress. This initiates unfolded protein response (UPR) to maintain homeostasis in the ER (Minamino et al., 2010). However, sustained UPR can cause cell death. Consequently, expression of GRP78 is increased acting as a quality control system.

HSP60 is a chaperone protein that forms a complex with the chaperone protein, HSP10 to promote protein folding. HSP60 mainly exists in the mitochondria, but can also be distributed within the cytoplasm, cell membrane and extracellular matrix (Rizzo et al., 2011).

Small HSPs are a group of proteins which are small size (12–42 kDa) and are present in the cytoplasm and nucleus. Small HSPs include HSP20, HSP27, heme oxygenase-1 (HO-1), and αB-crystallin (CRYAB). HSPs are involved in the regulation of anti-oxidants, anti-apoptosis, muscle contraction and cell motility, which can prevent irreversible aggregation of damaged proteins in an ATP-independent manner and protect cells under unfavorable conditions (Mymrikov et al., 2011).

## Function of HSPs in HF

**FIGURE 1:**
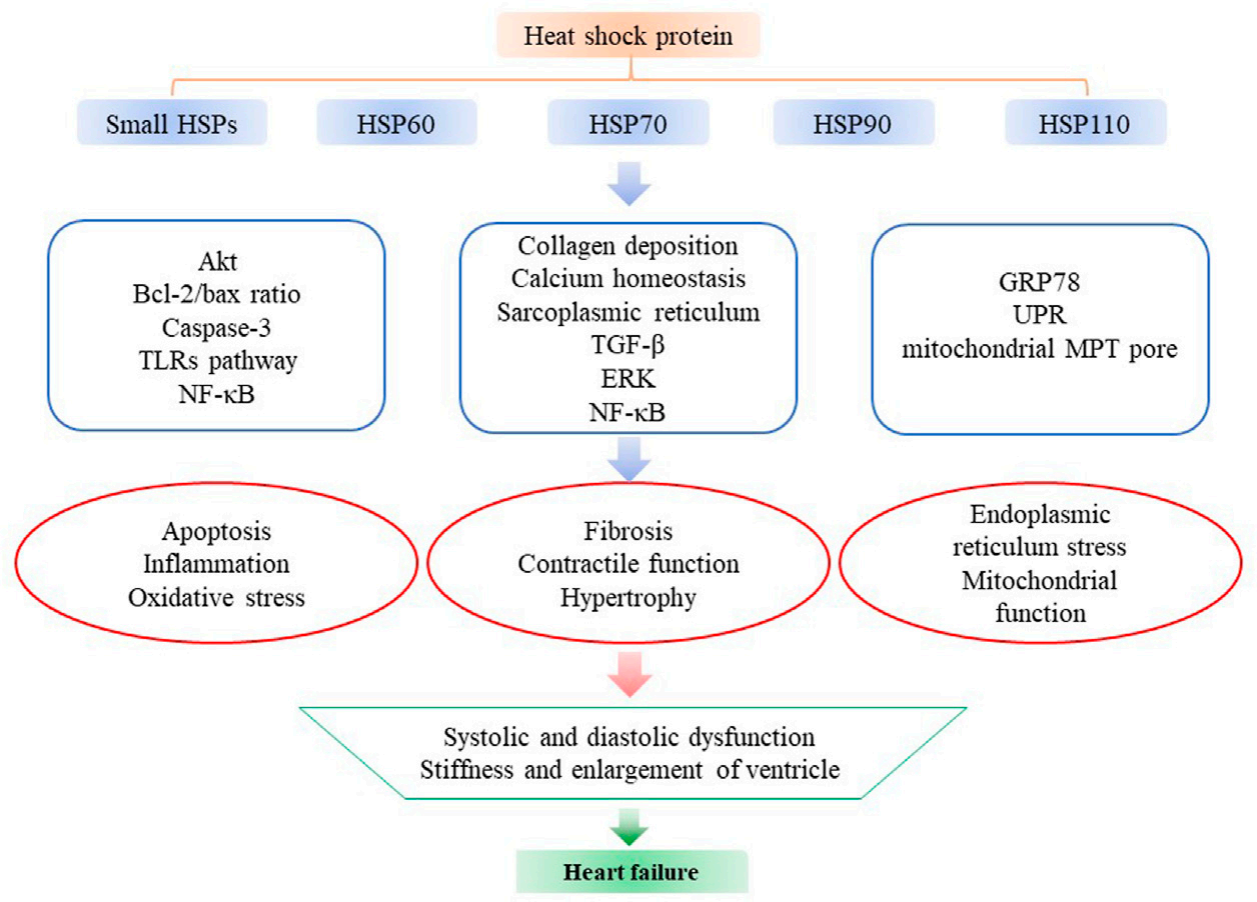
The functions of heat shock proteins in HF. Small HSPs (HSP27, HSP20, HO-1), HSP60, HSP70, HSP90, and HSP110 are the most studied HSPs in HF. They can affect apoptosis, inflammation, oxidative stress, fibrosis, contractile function, hypertrophy, ER stress and mitochondrial function by regulating multiple pathways like Akt, caspase-3, ERK and various cellular functions like ER and mitochondria in the progression of HF, including modulating the systolic and diastolic function and the stiffness and enlargement of ventricle.

HSPs participate in a wide range of biological activities, can contribute to intracellular homeostasis in cells and counteract pathological factors. Previous studies have investigated changes in the expression of HSPs in HF and the effects of overexpressed/deficient HSPs in HF. In this review, we have summarized recent advances in functions of HSPs in HF (Figure 1; Table 1).

**TABLE 1:** The functions of heat shock proteins in heart failure.

HSP family	Function	Model	Protective/adverse effects of HSPs in HF	Ref
HSP110	HSPA4 deletion leads to cardiac hypertrophy and fibrosis	HSPA4 knockout mice that subjected to transverse aortic constriction and volume overload	protective	Mohamed et al. (2012)
HSP90	Inhibition of HSP90 improves cardiac function	Rats that subjected to CAL.	adverse	Tamura et al. (2019)
	HSP90 can regulate cardiac hypertrophy and collagen deposition	Mice overexpression of HSP75 (a member of HSP90 family located in the mitochondria)	adverse	Lee et al. (2010); Datta et al. (2015); Tamura et al. (2019), Zhang et al. (2011)
	HSP90 facilitates regulation of Raf/Mek/ERK, TGF-β and NF-κB pathways in cardiac hypertrophy	Mice overexpression of HSP75	adverse	Lee et al. (2010); Datta et al. (2015); Tamura et al. (2019), Zhang et al. (2011)
	Interacts with TGFβ receptor-II and exerts profibrotic effect	Rats that subjected to renal artery ligation; Cardiac fibroblasts that subjected to Ang II and Celastrol	adverse	Datta et al. (2015)
	Interacts with IKK complex, leads to NF-κB activation	Ang II-induced cardiac myocytes	adverse	Lee et al. (2010)
	HSP75 downregulates TAK, p38, JNK, and Akt phosphorylation levels	Cardiac-specific HSP75 transgenic mice that subjected to aortic banding	protective	Zhang et al. (2011)
HSP70	Maintains cardiac contractility and calcium handling	HSP70-konckout mice that subjected to I/R	protective	Kim et al. (2006)
	Inhibits p53 activation and its downstream bax, caspase-3 and caspase-9	DOX-induced HSP70 overexpress transgenic mice	protective	Naka K et al. (2014)
	Does not improve cardiac function in failing hearts with atrial fibrillation	Cardiac-specific MURC mice and MURC-HSP70 mice	Undetermined	Bernardo et al. (2015)
	Both intracellular and extracellular HSP70 regulates myocardial hypertrophy, cardiac dysfunction and cardiac fibrosis	Mice that subjected to abdominal aortic constriction (AAC)	adverse	Cai et al. (2010)
	Extracellular HSP70 promotes cardiac hypertrophy and fibrosis	Mice that subjected to abdominal aortic constriction (AAC)	adverse	Cai et al. (2010)
	Extracellular HSP70 activates TLR2 signaling	TLR2/4 knockout mice that subjected to transverse aortic constriction (TAC). Mice treated with anti-HSP70 antibody and DOX.	adverse	Higashikuni et al. (2013), Liu et al. (2019)
	GRP78 attenuates ER stress and cell death	Neonatal cardiomyocytes that subjected to MG132, epoxomicin or tunicamycin	protective	Fu et al. (2008)
HSP60	Inhibits caspase-3 activation, interacts with bax and bcl-x	HSP60 and HSP10 overexpressed myocytes that subjected to DOX.	protective	Shan et al. (2003)
	Interacts with bak and bax in cytoplasm	Myocytes that subjected to antisense phosphorothioate oligonucleotide to reduce HSP60	protective	Kirchhoff et al. (2002)
	Maintains mitochondrial homeostasis function	Cardiac-specific HSP60 knockout mice	protective	Fan et al. (2020)
	Extracellular HSP60 activates TLR4 and triggers inflammation	Cardiomyocytes of rats that subjected to LAD.	adverse	Liu et al. (2015)
small HSPs—HSP27	Enhances the SOD activity, increases cell survival	DOX-induced cardiac H9c2 cells and mouse embryonic fibroblasts	protective	Turakhia et al. (2007)
	Improves cardiac function, suppresses oxidative stress and decreases apoptosis	DOX-induced cardiac specific-overexpressed HSP27 mice	protective	Liu et al. (2007a)
	Increases phosphorylation of Akt and GSK-3β, decreases NF-κB activation	LPS-induced cardiac-specific expression of Hsp27 and H9c2	protective	You et al. (2009)
	Preserves mitochondrial function	Rats that subjected to coronary artery ligation (CAL)	protective	Marunouchi et al. (2013c), (Marunouchi et al. (2014)
	Interacts with SIRT1; increases p53 acetylation and bax when be downregulated	Transfected H9c2 cells that subjected to DOX.	protective	Zhang et al. (2016a)
	High level HSP27 causes reductive stress and develops cardiac dysfunction	HSP27 transgenic mice	adverse	Yu et al. (2015), Zhang et al. (2010)
	Binds to p53 and increases bax contents	DOX-induced HSF-1 knockout mice	protective	Vedam et al. (2010)
small HSPs—HSP20	HSP20 reverse cardiac remodeling, fibrosis and hypertrophy	ISO-induced cardiac-specific overexpressed HSP20 mice and H9c2 cells	protective	Fan et al. (2006)
	Ameliorates cardiac dysfunction and suppresses ASK1 activation	ISO-induced cardiac-specific overexpressed HSP20 mice and H9c2 cells	protective	Fan et al. (2006)
	Inhibits NF-κB activation and caspase-3 activity	LPS-induced Ad. HSP20-AS-infected rat cardiomyocytes	protective	Wang et al. (2009)
	Preserves Akt activation, improves cardiac function	DOX-induced cardiac-specific overexpressed HSP20 mice	protective	Fan et al. (2008)
small HSPs—HO-1	Reduces oxidative stress and preserves mitochondrial function	Cardiac-specific HO-1 transgenic mice that subjected to CAL.	protective	Wang et al. (2010b)
	Preserves cardiac function	AAV-human HO-1 treated rats that subjected to LAD.	protective	Liu et al. (2007b)
	Increases Akt activation and decreases apoptosis	Ang II-induced myocytes that transfected with human HO-1	protective	Foo et al. (2006)
	Exerts either protective or detrimental effect	Cardiac-specific HO-1 mice that subjected to either TAC or ISO.	Dual	Allwood et al. (2014)

### HSP110

HSPA4 is a member of the HSP110 family that acts as a nucleotide exchange factor for HSP70 chaperones. Expression of HSPA4 was significantly elevated in hearts of mice subjected to TAC (Mohamed et al., 2012). HSPA4 is essential in ensuring proper folding of proteins and maintaining homeostasis in cardiomyocytes. Deletion of HSPA4 accelerates cardiac hypertrophy and fibrosis (Mohamed et al., 2012).

### HSP90

Expression of HSP90 was decreased in animals treated with fluoride (Panneerselvam et al., 2017), and no significant change was observed after CAL in comparison with control group (Tanonaka et al., 2001a), whereas expression of HSP90 increased in patients with DCM (Kapustian et al., 2013). DCM alters distribution of HSP90 in cells: mitochondrial HSP90 content was increased in the left ventricular myocardium of individuals with DCM (Kapustian et al., 2013). HSP90 can have a detrimental effect on HF and cardiac hypertrophy. Inhibiting functional expression of HSP90 can attenuate cardiac hypertrophy and reduce collagen deposition. HSP90 facilitates regulation of Raf/Mek/ERK, transformation of growth factor-β (TGF-β) and NF-κB pathways in cardiac hypertrophy which are either induced by MI or pressure overload (Lee et al., 2010; Datta et al., 2015; Tamura et al., 2019). Mice with cardiac-specific overexpressed HSP75 (a member of HSP90 family located in the mitochondria) may attenuate hypertrophy and fibrosis in response to pressure overload. Protection depends on the inhibitory effect of HSP75 in regulating MAPK and Akt pathways by reducing phosphorylation of p38, JNK and Akt (Zhang et al., 2011).

### HSP70

Previous studies have proven the protective function of HSP70s in HF. Expression of HSP70 in HF varies with models. Levels of intracellular HSP70 were elevated in patients with HF of arrhythmogenic right ventricular cardiomyopathy (ARVC), ischemic cardiomyopathy (ICM) and DCM (Wei et al., 2009). Nonetheless, expression of HSP70 remained unchanged at 8 w after CAL in rats in comparison with the control group, accompanied by decreased cardiac contractility and function. HSP70 was not induced even under heat stress (Tanonaka et al., 2001b; Tanonaka et al., 2001c). Myocardial dysfunction of CAL-induced HF was partially due to impaired induction of HSP70 and the mechanisms can be elucidated as follows: 1) total expression of HSF-1 is enhanced in CAL-induced HF rat model, whereas phosphorylated HSF-1 at ser303 is accumulated in the cytoplasm and fails to translocate to the nucleus thereby becoming incapable of inducing HSP70 (Marunouchi et al., 2013a), 2) interaction of HSP90 and HSF-1 is enhanced in the cytoplasm hindering nuclear translocation of HSF-1 (Marunouchi et al., 2013b), 3) downregulated mitochondrial aldehyde dehydrogenase2 (ALDH2) and the upregulated 4-hydroxy-2-nonenol (4-HNE) suppress expression of HSP70 in response to hypoxia, and this process is independent of HSF-1 (Sun et al., 2014). HSP70 can inhibit apoptosis and enhance tolerance to harmful stimuli to protect the heart from further damage. HSP70 knockout mice are more susceptible to ischemia/reperfusion (I/R) injury and more likely to develop myocardial hypertrophy resulting in decreased Ca^2+^ in the sarcoplasmic reticulum, damaged myocardial contractility, activation of JNK, p38, Raf-1 and extracellular regulated protein kinases (ERKs) pathways (Kim et al., 2006). Overexpressed HSP70 can protect mice from HF induced by DOX by inactivating p53 and its downstream bax, caspase-3 and caspase-9 (Naka K et al., 2014). However, long-term overexpression of HSP70 does not mitigate cardiac dysfunction and reverses remodeling in failing hearts with atrial fibrillation (AF). This indicates that HSP70 can be beneficial during acute cardiac condition but it cannot adequately inhibit chronic stimuli (Bernardo et al., 2015; Bernardo et al., 2016).

Intracellular HSP70 and extracellular HSP70 have differential effects on pressure overload-induced HF. Inhibition of HSP70 expression (both intracellular and extracellular) through inactivation of HSF-1 can promote myocardial hypertrophy and cardiac dysfunction but ameliorate cardiac fibrosis; functional inhibition of extracellular HSP70 using anti-HSP70 attenuates cardiac hypertrophy and fibrosis (Cai et al., 2010). Results of a study indicated the protective effect of intracellular HSP70 in cardiac function, and that the potential mechanism of anti-HSP70 lies in its inhibitory effect on ERK and p38 pathway through neutralization of extracellular HSP70. Concentration of plasma HSP70 was increased in both TAC-induced pressure overload and DOX-induced HF mice models. Extracellular HSP70 activates TLR2/NF-κB pathway, triggers inflammation and causes cardiac hypertrophy and fibrosis (Higashikuni et al., 2013; Liu et al., 2019). Furthermore, anti-HSP70 antibodies attenuate cardiac dysfunction induced by TAC or DOX by blocking extracellular HSP70-mediated activation of TLR2 pathway (Higashikuni et al., 2013; Liu et al., 2019). Plasma HSP70 was significantly increased in patients with HF and ARVC, ICM or DCM (Genth-Zotz et al., 2004; Gombos et al., 2008; Wei et al., 2009). Plasma HSP70 can be an independent prognostic biomarker for early diagnosis and is suitable for predicting long-term survival of patients with HF (Li et al., 2013a; Jenei et al., 2013).

Stress-induced UPR in the endoplasmic reticulum plays crucial role in the development and progression of HF (Minamino et al., 2010). Increased expression levels of GRP78, a marker of ER stress can also be an indicator of impaired UPR during progression of HF (Okada et al., 2004; Dally et al., 2009; Sawada et al., 2010). However, overexpressed GRP78 has a protective function in myocytes (Fu et al., 2008).

### HSP60

Unlike other HSPs, expression of HSP60 was elevated at 8w after CAL, and elevated HSP60 expression was driven by loss in the transcriptional activity of NF-κB for heat shock factor-1 (HSF-1) and failure to induce HSP72 in CAL-induced HF (Tanonaka et al., 2001a; Toga et al., 2007; Wang et al., 2010a). In addition, HF and DCM induced mitochondrial translocation of HSP60 (Sidorik et al., 2005; Lin et al., 2007). The potential protective mechanisms of HSP60 in the myocardium are involvement in anti-apoptosis and preservation of mitochondrial function. HSP60 can increase b-cell lymphoma-2 (bcl-2)/bcl-2-associated x (bax) ratio, inhibit caspase-3 and poly (ADP-ribose) polymerase (PARP) (Kirchhoff et al., 2002; Shan et al., 2003). HSP60 deletion causes HF in mice and impairs mitochondrial protein homeostasis (Fan et al., 2020). HSP60 transfers to the plasma and plasma membrane in HF, and its surface translocation is highly associated with apoptosis (Lin et al., 2007). Extracellular HSP60 can trigger toll-like receptor4 (TLR4) pathway and induce inflammatory response (Liu et al., 2015). The plasma HSP60 is positively correlated with occurrence of adverse cardiac events in both acute and chronic HF, implicating its potential of being a biomarker of HF (Niizeki et al., 2008; Zhang et al., 2008; Bonanad et al., 2013).

### Small HSPs—HSP27

HSP27 (also called HSP25 in murine) is involved in numerous cellular functions; it can counteract apoptosis and oxidative stress, and inhibit cardiac remodeling and dysfunction of a failing heart (Liu et al., 2007a; Turakhia et al., 2007; You et al., 2009; Marunouchi et al., 2013c; Marunouchi et al., 2014). Expression levels of HSP27 are increased in failing hearts, and this is induced by doxorubicin (DOX) and fluoride (Vedam et al., 2010; Panneerselvam et al., 2017) as a response to harmful stimuli. HSP27 may possibly have a dual effect on HF; it not only acts as an antioxidant to protect the heart from damages and improve cardiac function (Liu et al., 2007a; Turakhia et al., 2007; You et al., 2009), but also augments injury in a failing heart (Vedam et al., 2010; Zhang et al., 2010; Yu et al., 2015). Overexpression and phosphorylation of HSP27 counteracts the cardiotoxic effect of DOX, mitigates cardiac dysfunction in dilated cardiomyopathy (DCM) and congestive HF (Liu et al., 2007a; Turakhia et al., 2007). Cardiac-specific overexpressed HSP27 enhances phosphorylation of serine/threonine kinase (Akt), attenuates activation of glycogen synthase kinase-3β (GSK-3β) and nuclear factor kappa-B (NF-κB) to ameliorate cardiac dysfunction induced by lipopolysaccharide (LPS) (You et al., 2009). Expression and phosphorylation of HSP27 in the cytoplasm and mitochondria increased at 2w after coronary artery ligation (CAL) but decreased in the mitochondria at 8 w. This indicates that mitochondrial HSP27 and phosphorylated HSP27 significantly contribute to mitochondrial function in HF (Marunouchi et al., 2013c; Marunouchi et al., 2014). The co-chaperones of HSP27 alter its function. Downregulation of HSP27 hinders interaction of silent information regulator1 (SIRT1)-p53 and endowed p53 acetylation, augmenting apoptosis in DOX-induced H9c2 cells (Zhang et al., 2016a). However, inducible HSP27 can be pro-apoptotic by binding to and transactivating p53 resulting in loss of cardiomyocytes in HF (Vedam et al., 2010). Moderate level of HSP27 is beneficial, whereas higher levels of HSP27 can induce reductive stress and aggravate cardiomyopathy (Zhang et al., 2010; Yu et al., 2015). Plasma HSP27 is regarded as a novel candidate biomarker for diagnosing chronic HF and an independent predictor of HF- related mortality (Liu et al., 2016a; Traxler et al., 2017).

### Other Small HSPs

Other HSPs are also involved in the pathophysiology of HF. HSP20 has anti-apoptotic and anti-oxidative effects in cardiomyocytes which improve cardiac function. HSP20 can reverse cardiac remodeling, fibrosis and hypertrophy induced by isoproterenol (ISO) by inhibiting apoptosis signal regulating kinase1 (ASK1)/Jun N-terminal kinase (JNK)/p38 pathways (Fan et al., 2006). HSP20 decreases activity of NF-κB to attenuate apoptosis and myocardial dysfunction induced by LPS (Wang et al., 2009). HSP20 maintains activity of Akt signaling pathway and suppresses oxidative stress to alleviate damage of DOX (Fan et al., 2008). Expression of HO-1 was elevated at both protein and mRNA levels in the right-sided HF and post-myocardial infarction (MI) HF (Raju et al., 1999; Wang et al., 2010b). HO-1 induces anti-oxidant and anti-apoptotic effects, and enhances tolerance to HF. HO-1 can attenuate cardiac hypertrophy, fibrosis, oxidative stress, mitochondrial MPT pore (mPTP) opening and promote angiogenesis to preserve left ventricular function and attenuates remodeling of post-MI HF (Liu et al., 2007b; Wang et al., 2010b). Overexpressed HO-1 activates Akt pathway to reduce apoptosis in myocytes which is induced by angiotensin II (Ang II) (Foo et al., 2006). However, the protective role of HO-1 seems to depend on the type of stimulation. HO-1 significantly attenuated ISO-induced cardiac dysfunction, fibrosis and hypertrophy, but was detrimental in aging and transverse aortic constriction (TAC) models (Allwood et al., 2014).

In conclusion, HSPs make significant contributions in HF and most HSPs can exhibit protective effects whereas a few HSPs may accelerate damage based on a specific condition. Functions of HSPs seem to vary with their location: intracellular HSPs exhibit anti-apoptotic, anti-inflammatory and anti-oxidative effects, whereas extracellular HSPs are on the contrary. Moreover, HSPs modulate several signaling pathways to initiate biological effects. Consequently, regulation of the expression of HSPs is a promising treatment for HF.

## TCM Regulates Expression of HSPs

**FIGURE 2:**
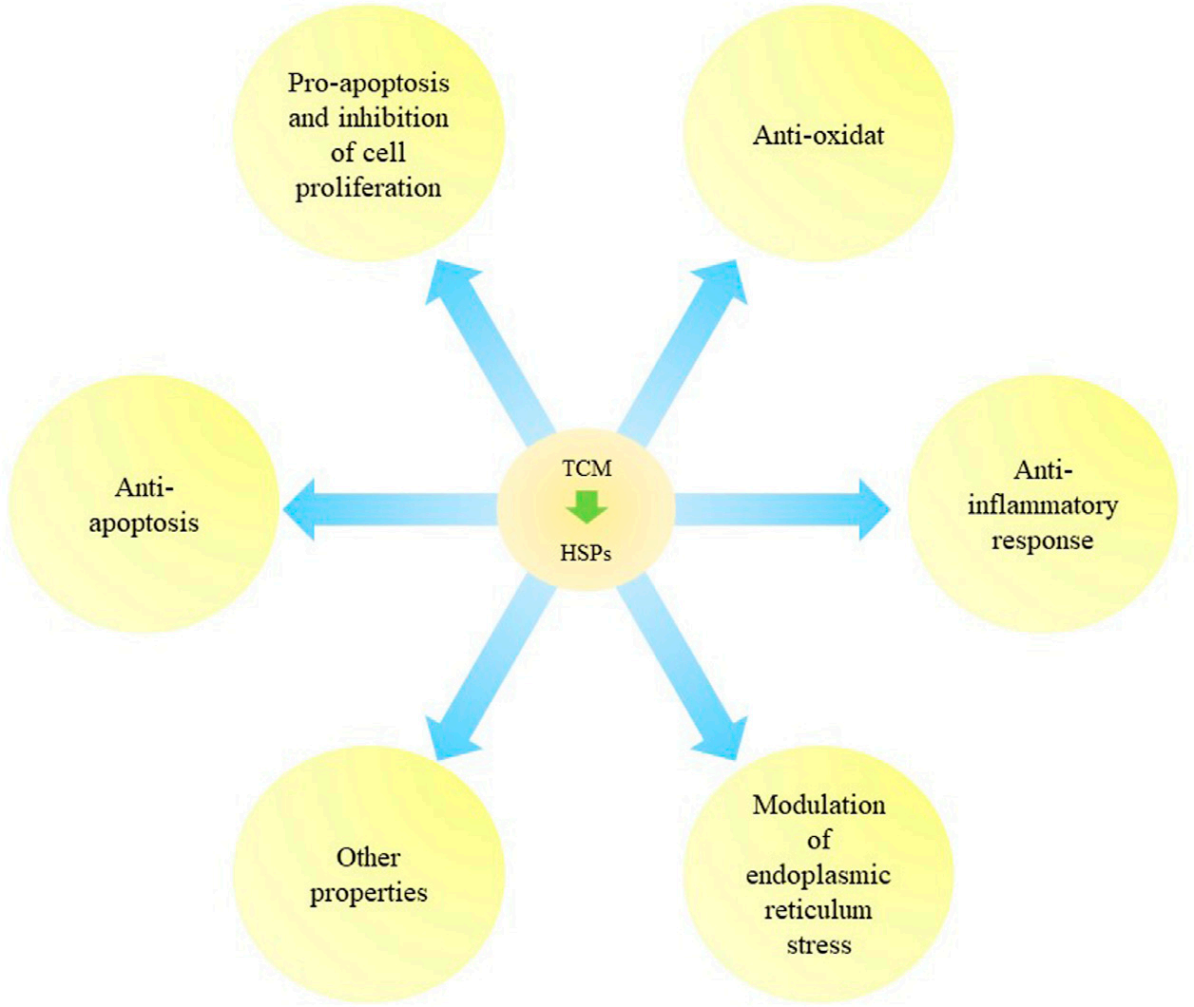
The regulation of Traditional Chinese Medicine on heat shock proteins. Traditional Chinese medicine (TCM) can exert various biological functions like anti-apoptosis, pro-apoptosis and inhibition of cell proliferation, anti-oxidant, anti-inflammatory response, modulation of ER stress and other properties via regulating HSPs.

TCM can regulate expression of HSPs to initiate anti-apoptotic, pro-apoptotic and anti-inflammatory responses. TCM can be used as anti-oxidants and for modulating ER stress in cancer, diseases of the nervous system, ischemic diseases, hepatopathy, gastroenteropathy and uterine diseases. Regulatory effects of TCM on HSPs are summarized and listed in Figure 2 and Table 2.

**TABLE 2:** The regulation of Traditional Chinese Medicine on heat shock proteins.

Property	TCM or active ingredients	Targets	Model	Ref
Anti-apoptosis	Resveratrol	↑ HSP27	Ultraviolet B-treated HaCaT cells	Zhou et al. (2018)
	Hydroxysafflor yellow A, extract of *Carthamus tinctorius L*	↓ phosphorylation of HSP27 at ser 78	Heat stress-induced neural stem cells	Li et al. (2019a)
	Zanthoxylum bungeanum Maxim	↑ HO-1	D-Galactose-Induced Aging Mice	Zhao et al. (2020)
	Icariin	↑ HSP70	Calvaria osteoblasts of rats	Qian et al. (2018)
	EGb761, extract of Ginkgo biloba leaves	↑ HSP70 and GRP78	Aβ_1-42_ oligomer-induced SH-SY5Y cells	Liu et al. (2016b)
	Ginsenosides Rg1 and Rb1 (extracts of *panax notoginseng*)	↑ HSP70	MCAO mice	Zeng et al. (2014)
	Tanshinone IIA	↑ HSP70	Rats that subjected to spinal I/R injury	Zhang et al. (2012)
	Gualou Guizhi decoction	↑ HSP70	Rats that subjected to MCAO.	Nan et al. (2020)
	Qinghuobaiduyin formula	↑ HSP70	Rats that subjected to burn injury	Zhu et al. (2013)
	Xiaotan Tongfu granule	↑ HSP70	Rats that subjected to cold-restraint model	Yan et al. (2013)
Pro-apoptosis and inhibit cells proliferation	Barbaloin, extract of *Aloe barnadensis Miller* leaves	↓ HSP27	NSCLC cell line A549	Zhang et al. (2017)
	Lariciresinol	↓ HSP27	HepG2 cells	Ma et al. (2016)
	Bufalin	↓ HSP27	Pancreatic cancer cells	Li et al. (2014)
	Tanshinone IIA, extract of *Salvia miltiorrhiza*	↑ phosphorylation of HSP27 at ser 82	Human gastric cell line AGS	Yin et al. (2020)
	Curcumin, extract of *Curcuma longa*	↓ HSP27	Human colon cancer HCT-8 and HCT-8/5-FU (5-FU-resistant cell line)	He et al. (2019a)
	Synergistic application of triptolide and celastrol	↓ HSP27, HSP70 and HSP90	Human cancer cell lines and human normal embryonic kidney cell line HEK293T	Jiang et al. (2015)
	Homogeneous *Schisandra chinensis* polysaccharide-0-1	↓ HSP90	HepG2 cells	Chen et al. (2016)
	Patrinia heterophylla	↓ HSP90	Leukemia K562 cells	Wei et al. (2012)
	Platycodin D, extract of *Platycodonis Radix*	↓ Hsp90/Cdc37 interactions	Human lung cancer cells	Li et al. (2017)
Anti-oxidative property	Zanthoxylum bungeanum Maxim	↑ HO-1	D-Galactose-Induced Aging Mice	Zhao et al. (2020)
	Celastrol (extract of *Tripterygium wilfordii* Hook)	↑ HO-1 and HSP70	Lipopolysaccharide (LPS)-induced rats	Wang et al. (2015a)
	Protopanaxtriol	↑ HO-1 and HSP70	Rats that subjected to 3-nitropropionic acid	Gao et al. (2015)
	Radix Bupleuri extract	↑ HO-1, ↓ HSP70	H_2_O_2_-induced Tilapia	Jia et al. (2019)
	Water extract and ethanol extract of *Cordyceps cicadae*	↑ HO-1	Cisplatin-induced mouse	Deng et al. (2020)
	Diethyl blechnic, a compound isolated from Danshen	↑ HO-1	LPS-induced RAW264.7 cells	He et al. (2019b)
Anti-inflammation	Celastrol, extract of *Tripterygium wilfordii* Hook	↑ HO-1 and HSP70	LPS-induced rats	Wang et al. (2015a)
	Radix Bupleuri extract	↑ HO-1, ↓ HSP70	H_2_O_2_-induced Tilapia	Jia et al. (2019)
	Momordica grosvenori	↑ HO-1	LPS-induced RAW264.7 cells	Li et al. (2019b)
	Liquiritigenin and liquiritin	↓ extracellular release of HSP60	Monocrotaline-induced Hepatic sinusoidal obstruction syndrome in rats	Huang et al. (2019)
	Rhodiola rosea L. root and rhizome extract	↑ HSP70	CRH-stimulated BV2 microglial cells	Borgonetti et al. (2020)
	Xiaotan Tongfu granule	↑ HSP70	Rats that subjected to cold-restraint model	Yan et al. (2013)
	Emodin-8-O-glucuronic acid, isolated from qinghuobaiduyin decoction	↑ HSP70	LPS-stimulated raw 264.7 cells	Wang et al. (2016)
Modulate ER stress	Bitter melon	↓ GRP78	Human colonic adenocarcinoma LS174T cells	Kunde et al. (2017)
	Gambogenic acid, a compound of *Garcinia hanburyi* HOOK	↓ GRP78	Human nasopharyngeal carcinoma cells	Su et al. (2019)
	Glycyrrhetinic acid, a component of *glycyrrhiza*	↑ GRP78	Human NSCLC cells	Zhu et al. (2015)
	Rhein, a compound of rhubarb	↓ GRP78	MCF-7 and HepG2 cells	Wang et al. (2015b)
	Xuefuzhuyu capsules	↓ GRP78	Rats subjected to hindlimb unload	Zhang et al. (2018b)
	Bushen Zhuangjin decoction	↓ GRP78	Tunicamycin induced-articular chondrocytes	Lin et al. (2015)
Others	Licorice, extract of Glycyrrhiza uralensis Fisch	↓ phosphorylation of HSP27, alters the interaction of HSP27 and actin	Oxytocin-induced uterine contraction	Yang et al. (2017a)
	Schisandrin B, isolated from a *Schisandra chinensis*	↑ HSP27 and HSP70	D-galactosamine-induced liver injury in mice	Gao et al. (2016)
	Combination use of ferulic acid, ligustrazine and tetrahydropalmatine	↓ HSP90	Endometriosis rats	Tang et al. (2014)
	Uncaria rhynchophylla	↓ HSP90	MPP^+^ -induced SHSY5Y cells and MPTP-induced mice	Lan et al. (2018)
	Zhenbao Pill	↑ HSP27	Rats that subjected to acute spinal cord injury	He et al. (2018)
	YangZheng XiaoJi formula	↓ phosphorylation of HSP27	Human gastric cancer, pancreatic cancer, ovarian cancer), lung cancer, breast cancer, prostate cancer, ovarian cancer cells	Owen et al. (2016)

### Anti-Apoptosis

Resveratrol inhibits apoptosis in ultraviolet B-treated HaCaT cells, and can upregulate HSP27 expression, increase bcl-2/bax ratio, and inhibit caspase-3 activity and p65 expression (Zhou et al., 2018). Hydroxysafflor yellow A is extracted from the flowers of *Carthamus tinctorius L.*; it can inhibit phosphorylation of p38 and HSP27 Ser78, and prevent apoptosis in heat stress-induced neural stem cells (NSCs) (Li et al., 2019a). Icariin upregulates HSP70 and serpin family F-1 (PEDF-1) to promote proliferation, calcium deposition and inhibits osteoblast apoptosis (Qian et al., 2018). Pretreatment with EGb761, an extract of *Ginkgo biloba* leaves can increase levels of HSP70 and GRP78 to reduce apoptosis and neurotoxicity in Aβ_1-42_ oligomer-induced SH-SY5Y cells (Liu et al., 2016b). Ginsenosides Rg1 and Rb1, extracts of *Panax notoginseng* increased HSP70 levels and restored the Akt/NF-κB signaling pathway in the hippocampus, causing neuroprotective effects against cerebral I/R (Zeng et al., 2014). Tanshinone IIA can attenuate spinal I/R injury and promote expression of HSP70 and bcl-2 (Zhang et al., 2012). Some formula can also be anti-apoptotic. Gualou Guizhi decoction increases expression of HSP70 in middle cerebral artery occlusion (MCAO) rat model and alleviates neuronal apoptosis by inhibiting PARP-1/apoptosis inducing factor (AIF) signaling pathway (Nan et al., 2020). Qinghuobaiduyin formula (contains extracts of *Astragalus membranaceus*, *Lonicera japonica*, *Scutellaria baicalenis Georgi*, *Ophiopogon japonicus* and *Rheum rhabarbarum*) increases HSP70 levels and induces anti-apoptotic effects on the intestinal mucosa following burn injury (Zhu et al., 2013). Granules of Xiaotan Tongfu promote cell proliferation, inhibit gastric mucosal cell apoptosis and local inflammation, and increase expression of HSP70 in rats with stress ulceration (Yan et al., 2013).

### Pro-Apoptosis and Inhibition of Cell Proliferation

Induction of apoptosis in cancer cells is vital and certain types of TCM can inactivate HSPs resulting in increased cell death. Barbaloin which is extracted from leaves of *Aloe barbadensis Miller*, inactivates p38 mitogen-activated protein kinase (MAPK)/HSP27 pathway, induces apoptosis and inhibits growth of human non-small cell lung cancer (NSCLC) cell line, A549 (Zhang et al., 2017). Lariciresinol downregulates HSP27 and initiates apoptosis in HepG2 cells (Ma et al., 2016). Bufalin induces apoptosis by partially targeting HSP27, eliminates anti-apoptotic effect of HSP27 in pancreatic cancer cells, and induces caspase-3 and caspase-9 (Li et al., 2014). Temporal treatment with tanshinone IIA (a diterpene quinone extract from *Salvia miltiorrhiza*) increases phosphorylation of HSP27 at Ser 82, and subsequent overexpression of HSP27 limits tanshinone IIA-induced cell death in gastric cells (Yin et al., 2020). Curcumin is a hydrophobic polyphenol derived from the rhizomes of *Curcuma longa*, which can inhibit cell proliferation and decrease expression of HSP27 at mRNA levels in human colon cancer (HCT)-8 and HCT-8/5-FU (5-FU-resistant cell line) (He et al., 2019a). Triptolide reduces protein levels of HSP27, HSP70 and HSP90 whereas celastrol increases protein levels of HSP27 and HSP70. Synergistic application of triptolide and celastrol can mitigate effect of increased HSP27 and HSP70, inhibit growth of cancer cells, and induce apoptosis in cancer cells (Jiang et al., 2015). Homogeneous polysaccharide-0-1 (SCP-0-1) from *Schisandra chinensis* induces mitochondrial apoptosis in human hepatocellular liver carcinoma, a mechanism involved in the downregulation of HSP90 and inhibition of Akt pathway (Chen et al., 2016). *Patrinia heterophylla*, a member of Valerianaceae family, inhibits expression of HSP90α to induce apoptosis in leukemia K562 cells (Wei et al., 2012). Platycodin D is a saponin isolated from *Platycodonis radix*, which can disrupt Hsp90/Cdc37 co-chaperone interactions without affecting ATPase activity of HSP90 and reduces Akt phosphorylation in human lung cancer cells (Li et al., 2017).

### Anti-Oxidant

*Zanthoxylum bungeanum* Maxim is a plant that can be used both as a condiment and as medicine. Its extracts in water and volatile oil can activate Akt/nuclear factor E2-related factor 2 (Nrf2)/HO-1 pathway to prevent cognitive dysfunction and hippocampal neuronal cell damage which are induced by D-galactose (Zhao et al., 2020). Celastrol is extracted from the root of *Tripterygium wilfordii* Hook, and it possesses anti-oxidant and anti-inflammatory effects which can attenuate cardiac iNOS, tumor necrosis factor-α (TNF-α), NF-κB and activity of caspase-3. Celastrol can also increase contents of HO-1 and HSP70 in the heart and aorta to prevent circulatory failure in sepsis (Wang et al., 2015a). Protopanaxtriol increases expression of HO-1 to induce anti-oxidative effect, relatively increases reactive oxygen species (ROS) and HSP70, and alleviates behavior disorders in 3-nitropropionic acid-induced rat model of Huntington’s disease (Gao et al., 2015). Pretreatment with extracts from *Radix bupleuri* can reverse increased HSP70 at mRNA levels in liver injury induced by H_2_O_2_. The primary beneficial effects of *Radix bupleuri* extracts of inhibiting oxidative stress is due to its role in enhancing Nrf2/HO-1 signaling pathway and inhibiting TLRs/MyD88/NF-κB signaling pathway (Jia et al., 2019). Water and t and ethanol extracts of *Cordyceps cicadae* increase production of Nrf2, HO-1 and other antioxidants, inhibit activation of NF-κB, attenuates oxidative stress and inflammation to prevent cisplatin-induced kidney injury (Deng et al., 2020). Diethyl blechnic, a compound isolated from *Salvia miltiorrhiza*, increases expression of Nrf2/HO-1 and inhibits TLR4/MyD88 signaling pathway to ameliorate oxidative stress in LPS-induced RAW264.7 cells (He et al., 2019b).

### Anti-Inflammatory Response

*Momordica grosvenori* attenuates phosphorylation of Akt1 pathway, increases expression of HO-1 to initiate anti-inflammatory effect on LPS-induced RAW264.7 cells (Li et al., 2019b). Liquiritigenin and liquiritin are two key compounds in *Glycyrrhizae radix et Rhizoma*, which have the ability to alleviate liver inflammatory injury. These compounds can prevent release of HSP60 to the extracellular matrix in monocrotaline-induced rat models and block exogenous HSP60-activated NF-κB in RAW264.7 cells (Huang et al., 2019). Root and rhizome extracts of *Rhodiola rosea* L. increase expression of HSP70 in corticotropin releasing hormone (CRH)-stimulated BV2 microglial cells, counteract neuroinflammatory effect and enhance cell survival (Borgonetti et al., 2020). Emodin-8-O-glucuronic acid, a compound isolated from qinghuobaiduyin decoction (TCM), increases expression of HSP70 to inhibit inflammatory cytokines in the LPS-stimulated Raw 264.7 cells (Wang et al., 2016).

### Modulation of Endoplasmic Reticulum Stress

The chaperone heat shock protein GRP78, together with C/-EBP homologous protein (CHOP) are commonly used as markers of endoplasmic reticulum (ER) stress. As an ER chaperone, GRP78 functions as a potent anti-apoptotic factor and confers drug resistance, whereas CHOP is a key initiating factor of ER stress-related cell death. Moreover, as a master of UPR in ER of normal cells, GRP78 force the unfolded proteins to refold or degrade by cellular degradation mechanisms. While under stress, the overexpression of GRP78 on the cell membrane mediates the vast amount of disordered proteins (Ibrahim et al., 2019).

Gambogenic acid is a component of Gamboge, a dry resin obtained from *Garcinia hanburyi* HOOK. f. (Guttiferae), which downregulates GRP78 and upregulates CHOP to induce apoptosis in poorly differentiated human nasopharyngeal carcinoma cells (Su et al., 2019). Glycyrrhetinic acid, a bioactive component of *glycyrrhiza*, upregulates GRP78 and CHOP to modulate ER stress and suppresses proliferation of human NSCLC cells (Zhu et al., 2015). Rhein, a compound of rhubarb can adequately induce GRP78 and inhibit expression of GRP78 induced by ER stress, disrupting the anti-apoptotic pathway in cancer cells (Wang et al., 2015b). Bitter melon ameliorates ER stress in epithelial cells of the colon thus decreasing expression of GRP78 and CHOP (Kunde et al., 2017). Capsules of Xuefu Zhuyu decrease expression of GRP78 and CHOP to alleviate ER stress. Capsules also attenuate loss of muscle mass and cross-sectional areas induced by hindlimb unloading (Zhang et al., 2018b). A decoction of Bushen Zhuangjin downregulates expression of GRP78 and inhibits ER stress to suppress tunicamycin induced-chondrocyte apoptosis (Lin et al., 2015).

### Other Properties

Licorice is derived from the roots and rhizomes of *Glycyrrhiza uralensis Fisch*, and it reduces levels of phosphorylated HSP27 at Ser15, altering interaction of HSP27 and actin, and it decreases actin polymerization to enhance spasmolytic effects in oxytocin-stimulated uterus (Yang et al., 2017a). Schisandrin B is isolated from *Schisandra chinensis* and it attenuates D-galactosamine-induced liver injury in mice. Hepatoprotective effect of schisandrin B is partially attributed to increased levels of HSP27 and HSP70 (Gao et al., 2016). Combined use of ferulic acid, ligustrazine and tetrahydropalmatine enhance downregulation of hypothalamus–pituitary–ovarian axis (HPOA), estrogen response element (ERE) pathway and expression of HSP90 in rat model of endometriosis (Tang et al., 2014). *Uncaria rhynchophylla* inhibits expression of HSP90 and activates Akt pathway to induce neuroprotective effect in mouse model of Parkinson’s disease (Lan et al., 2018). Zhenbao pills promote expression of HSP27, affect Treg cell differentiation and ameliorate acute spinal cord injury in rats (He et al., 2018). YangZheng XiaoJi formula is able to inhibit phosphorylation of HSP27 and reduce migration of cancer cells (Owen et al., 2016).

## Therapeutic Functions of TCM in HF

TCM is widely distributed in nature and the various forms of TCM include signal herbs, formula, decoctions, capsules and others. Discovery and application of TCM is based on TCM theories. TCM with particular therapeutic effects have been applied in the treatment of HF in China for thousands of years. A systematic review has revealed that Shengmai (comprising herbs from *Panax ginseng*, *Ophiopogon japonicus* and *Schisandra chinensis*) improves ejection fraction, cardiac output, cardiac index, left ventricular end-systolic volume and myocardial contractility (Zhou et al., 2014). Clinical studies have mostly been conducted by the Chinese and recent studies come to emphasize a uniform standard.

Studies have summarized the commonly prescribed herbs for treating different HF syndromes are as follows: Radix aconiti carmichaeli (Fuzi), Atractylodes (Baizhu), Cassia twig (Guizhi), Dried ginger (Ganjiang), Radix pseudostellariae (Taizishen), Radix astragali (Huangqi), Codonopsis pilosula (Dangshen), Ginseng (Renshen), Panax notoginseng (Sanqi), Chinese angelica (Danggui), Safflower (Honghua), Ligusticum wallichii (Chuanxiong), Salvia miltiorrhiza (Danshen), Red paeony root (Chishao), Peach kernel (Taoren), Hawthorn (Shanzha), Semen lepidii (Tinglizi), Alisma (Xieze), Poria cocos (Fuling); Radix Ophiopogonis (Maidong), Fructus schisandrae (Wuweizi), Radix rehmanniae (Shengdi), Pinellia (Banxia), Trichosanthes Kirilowii (Gualou), Dried tangerine or orange peel (Chenpi), and Scallions white (Xiebai), etc (Wang et al., 2017a). Moreover, there are several most commonly prescribed formulae that have been proven effective clinically for the treatment of HF. These decoctions prescribed by physicians include: Zhenwu tang, Shengmai san, Baoyuan tang, Xuefuzhuyu tang, Tinglidazaoxiefei tang, Danshen yin, and Taohongsiwu tang etc. Meanwhile, several Chinese patent drugs have been successfully produced by standardized procedures and are widely used in health care industry. Drugs in the form of capsules or pills include: Qishenyiqi dripping pill (QSYQ), Fufang danshen dripping pill, Danqi pill (DQP), Qili qiangxin capsule, and Shengmai capsule, etc. The produced injections include: Shenmai injection, Shengmai injection, Huangqi injection, Shenfu injection, and Danhong injection, etc (Jian, 2002). Among these patent medicine above, a randomized clinical trial indicates QSYQ could promote left ventricular function, increase exercise capacity and reduce re-admission rate (Hou et al., 2013; Shang et al., 2013). A clinical trial of Qili qiangxin capsule demonstrated superior performance in comparison to the placebo in terms of NYHA functional classification, 6-min walking distance, LVEF and quality of life (Li et al., 2013b). The underlying mechanisms includes regulating TGF-β1 in the progression of fibrosis (Zhang et al., 2016b), or modulates the expressions of collagen I (Col I), collagen III (Col III), matrix metalloproteinase-2 (MMP-2), and MMP-9, which are the main contributors to extracellular matrix remodeling (Zhang et al., 2015).

I/R injury in myocardial infarction is an important inducing or exacerbating factor for acute HF. The underlying mechanisms of TCM in the treatment of HF include anti-fibrosis, anti-inflammation, anti-oxidant, anti-apoptosis, pro-angiogenesis effects and regulation of metabolism, thus directly mitigate the I/R injury or indirectly reducing the adverse cardiac remodeling which could induce or exacerbate HF. For example, dioscin attenuates apoptosis and oxidative stress by regulating bcl-2/bax ratio and SOD (). Shensong Yangxin and Sini Tang (comprising *Aconitum carmichaelii Debeaux*, *Cinnamomum cassia* (L.) J. Presl, *Zingiber officinale Roscoe* and *Glycyrrhiza uralensis* Fisch. ex DC.) can enhance cardiac function by suppressing cardiac collagen hyperplasia in rabbits and TGF-β1 expression in MI-induced rat models (Liu et al., 2014; Dang et al., 2016).

TCM is usually used together with western medicine to treat HF. The multiple effects of TCM can counteract adverse effects of pharmacological treatment, making it a potential therapeutic option. However, application of TCM is limited because of lack of large-scale multi-center clinical trials and experiments. Therefore, further research on the mechanism of TCM in treating HF is necessary to enhance its applicability worldwide.

### TCM Regulates Expression of HSPs in HF

**FIGURE 3:**
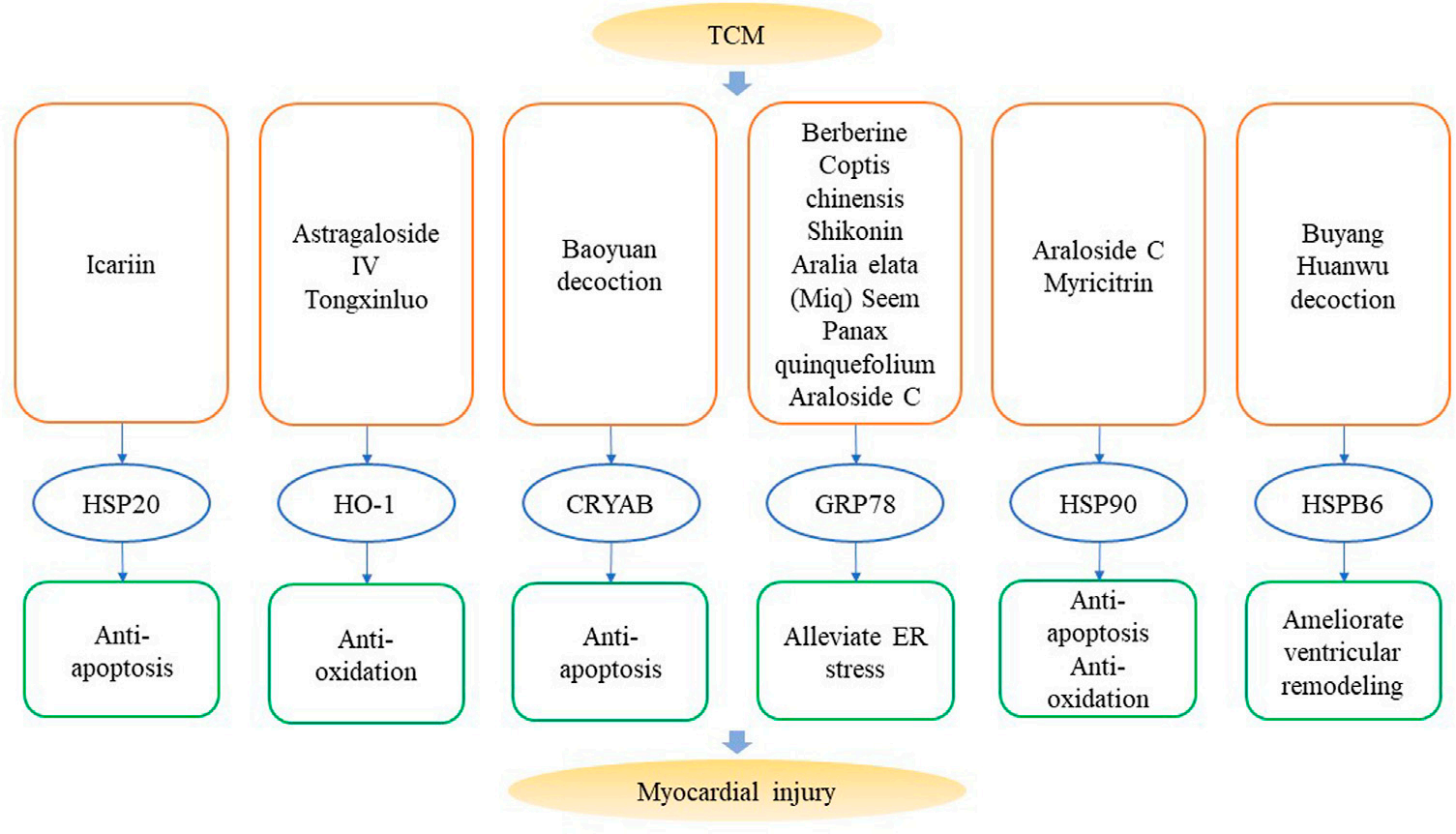
Traditional Chinese Medicine that target heat shock proteins in myocardial injuries. Components like icariin, astragaloside IV, berberine and decoctions like Baoyuan decoction and Buying Huanwu decoction can alleviate myocardial injury via anti-apoptosis, anti-oxidation, reducing ER stress and cardiac remodeling by regulating the expression of HSPs.

Based on the functions of HSPs in HF, regulation of HSPs and the protective effects of TCM in treating HF, it can be hypothesized that TCM regulate HSPs to enhance therapeutic effects on HF. A fraction of TCM has been proven to regulate HSPs in the myocardium and protect the heart from fibrosis, remodeling and hypertrophy. Functions of TCM which target HSPs in myocardial injuries are summarized in Figure 3 and Table 3.

**TABLE 3:** Traditional Chinese Medicine that target heat shock proteins in myocardial injuries.

Target	TCM	Function	Model	Ref
HSP20	Icariin	Upregulates HSP20 and suppresses apoptosis	H9C2 with H/R	Ren et al. (2018)
HO-1	Astragaloside IV, a component of *Astragalus membranaceus*	Activates Nrf2/HO-1 pathway, attenuates cardiac hypertrophy, improves left ventricular function and structure	Abdominal aortic constriction (AAC)-induced rats; Ang II-induced cardiomyocyte	Nie et al. (20192019)
	Tongxinluo	Upregulates cardiac expression of HO-1 and activates VEGF/Akt/eNOS pathway	TAC-induced HF in mice	Wang et al. (2014)
CRYAB	Baoyuan decoction	Activates CRYAB to inhibit apoptosis, rescues cardiac function	Rats that subjected to LAD; LPS-induced RAW 264.7 Cell; macrophage-conditioned media-stimulated H9C2 cells	Zhang et al. (2018a)
GRP78	Berberine, Coptis chinensis	Reduce apoptosis and ER stress, improve cardiac function and remodeling	Rats that subjected to LAD.	Liao et al. (2018)
	Shikonin	Inhibits α-SMA/collagen, TLR4/NF-κB signaling and ER stress pathway, decreases GRP78	ISO-induced mice and H9C2 cells	Yang et al. (2017b)
	Aralia elata (Miq) Seem	Alleviates ER stress-induced apoptosis, reduces GRP78	Rats that subjected to LAD.	Wang et al. (2018)
	Panax quinquefolium	Inhibits excessive ER stress and reduces GRP78	H/R-induced Ventricular cardiomyocytes	Wang et al. (2012)
	Araloside C	Attenuates ER stress-dependent apoptotic pathways	H/R-induced H9C2 cells. I/R-induced rat hearts	Du et al. (2018), Wang et al. (2017b)
HSP90	Araloside C	Reduces apoptosis by increasing HSP90 expression	H/R-induced H9C2 cells. I/R-induced rat hearts	Du et al. (2018), Wang et al. (2017b)
	Myricitrin	Increases expression of HSP90 to alleviate apoptosis and oxidative stress	H/R-induced H9C2 cells	Wang et al. (2017c)
HSPB6	Buyang Huanwu decoction	Increases the expression and phosphorylation of HSPB6, ameliorates ventricular remodeling	Rats with left anterior descending (LAD) artery ligation	Zhou et al. (2012)

On the one side, TCM could directly relieve HF by regulating HSPs and HSPs-mediated ER stress. Astragaloside IV is an active component of *Astragalus membranaceus*, can activate Nrf2/HO-1 pathway to protect the heart from hypertrophy and fibrosis (Nie et al., 20192019). Shikonin is extracted from the red-root gromwell, and it ameliorates ISO-induced myocardial damage, and cardiac hypertrophy by inhibiting α-smooth muscle actin (α-SMA)/collagen, TLR4/NF-κB signaling and ER stress pathways. Suppression of ER stress is reflected as decreased expression of GRP78 (Yang et al., 2017b). Tongxinluo is a TCM compound, which can increase cardiac expression of HO-1 and activate vascular endothelial growth factor (VEGF)/Akt/eNOS pathway to prevent TAC-induced HF in mice (Wang et al., 2014).

On the other side, as I/R injury in myocardial infarction is an important inducing or exacerbating factor for acute HF, TCM could also indirectly prevent HF pathogenesis by decreasing I/R injury and impeding fibrosis and cardiac remodeling in myocardial infarction. Berberine, a key active ingredient of *Coptis chinensi*s can improve cardiac function and remodeling, reduce apoptosis and ER stress (marked as decreased GRP78 and CHOP) in post-MI HF (Liao et al., 2018). Icariin suppresses apoptosis by reversing downregulation of HSP20 in H9c2 cells induced by hypoxia/reoxygenation (H/R) injury (Ren et al., 2018). Araloside C, a compound isolated from *Aralia elata* (Miq) Seem, icariin and *Panax quinquefolius* L. can ameliorate apoptosis and ER stress, reduce expression of GRP78 in myocytes induced by either I/R or tunicamycin (Wang et al., 2012; Zhang et al., 2013; Wang et al., 2017b; Du et al., 2018; Wang et al., 2018). In addition, Araloside C can increase expression of HSP90 and alleviate apoptosis in either H9c2 with H/R injury or rat with I/R injury (Wang et al., 2017b; Du et al., 2018). Myricitrin can also alleviate apoptosis and oxidative stress induced by H/R injury by increasing expression of HSP90, and the protective function of myricitrin partially depends on phosphatidylinositol 3-kinase (PI3K)/Akt pathway (Wang et al., 2017c). Buyang Huanwu decoction ameliorates I/R-induced ventricular remodeling by upregulating expression of HSPB6 and peroxiredoxin-6 (PRDX6), and downregulating atrial natriuretic factor (ANF) thereby decreasing activities of bax and caspase-3 (Zhou et al., 2012). Baoyuan decoction is a TCM formula composed of *astragalus, ginseng*, *liquorice* and *cinnamon*. It can activate CRYAB to inhibit apoptosis and enhance cardiac function in post-MI-induced HF (Zhang et al., 2018a). Scutellarin can alleviate apoptosis in H/R induced human cardiac microvascular endothelial cells (HCMECs) and increased expression of HSP60 might be a crucial factor for its protective effect (Shi et al., 20152015). Emodin restores activity of peroxisome proliferators-activated receptor-γ (PPAR-γ), eNOS phosphorylation, and interaction of HSP90/eNOS to alleviate H/R-induced injury in HAECs (Shou et al., 2018).

### Other Cardio-Protective Effects of TCM by Regulating HSPs

Besides HF and myocardial infarction, studies indicates TCM could also prohibit pathological process of atherosclerosis by regulating HSPs. Decoctions like Xiaoyaosan can inhibit expression of HSP27, HSP60 and HSP90, and promote interaction of HSP90 with glucocorticoid receptor (GR) and CD36 to prevent development of atherosclerotic vulnerable plaque in mouse model of atherosclerosis induced by high-fat food coupled with chronic stress (Fu et al., 2019). Ligustrazine increases NO production in human umbilical vein endothelial cells (HUVECs), downregulates intercellular cell adhesion molecule-1 (ICAM-1) and HSP60 expression levels to induce immunomodulatory effect on TNF-α-stimulated HUVECs (Wu et al., 2012). Baicalin increases HSP72 expression at both mRNA and protein levels in a cow’s mammary epithelial cells (CMECs) and inactivates NF-κB pathway to alleviate LPS-induced apoptosis (Yang et al., 2016). Catalpol, an extract of *Radix rehmannia*, inhibits homocysteine-induced apoptosis in the human aorta endothelial cells (HAECs) by suppressing Nox4/ROS/NF-κB pathway and GRP78/dsRNA-activated protein kinase–like endoplasmic reticulum kinase (PERK) pathway to alleviate ER stress (Hu et al., 2019).

## Conclusion and Perspectives

HF describes the terminal stage of multifarious heart diseases such as dilated cardiomyopathy, myocardial infarction and myocarditis. Pathogenesis of HF is characterized by cardiomyocyte apoptosis, oxidative stress, inflammation and mitochondrial dysfunction, all of which cause myocardial fibrosis and remodeling. HSPs have various functions, including regulation of apoptosis, anti-oxidant and anti-inflammation effects, and are capable of ameliorating cardiac dysfunction in HF. However, not all the HSPs are protective in HF, some HSPs exerts detrimental effects in HF progressive. Even some HSPs can modulate HF pathogenesis with dual effects. Thus, further studies are still required to explore accurate functions of HSPs in HF with different cell and molecular microenvironment. New treatment methods that focuses on the regulation of HSPs would have a promising application prospect in the prevention and treatment of HF.

TCM has been applied in the treatment of HF in China for thousands of years. Small sample clinical trials indicate the single compounds extracted from herbal medicine and formula, as well as patent medicine, are able to regulate HSPs in HF. Consequently, TCM is a potential therapeutic medium for modulating HSPs in HF and improving cardiac function. Studies on effects of various forms of TCM have confirmed the hypothesis that TCM alters expression of HSPs in HF but such studies are few. Thus, the application of TCM is limited in clinic because of lack of large-scale multi-center and randomized clinical trials. Therefore, further investigations on the effects of TCM in reliving HF by targeting HSPs are needed, and the underlying mechanisms involved in TCM regulating HSPs are also encouraged to be explored in future.

## Glossary

4-HNE: 4-hydroxy-2-nonenol
AAC: Abdominal aortic constriction;
ACEI: Angiotensin-converting enzyme inhibitors
AF: Atrial fibrillation
AIF: Apoptosis inducing factor
Akt: Serine/threonine kinase
ALDH2: Aldehyde dehydrogenase 2
ANF: Atrial natriuretic factor
Ang II: Angiotensin II
ARB: Angiotensin receptor blockers
ARNI: Angiotensin receptor neprilysin inhibitor
ARVC: Arrhythmogenic right ventricular cardiomyopathy
ASK1: Apoptosis signal regulating kinase1
bax: Bcl-2-associated x
bcl-2: B-cell lymphoma-2
CAL: Coronary artery ligation
CHOP: C/EBP-homologous protein
CMECs: Cow mammary epithelial cells
CRH: Corticotropin releasing hormone
CRYAB: αB-crystallin
DCM: dilated cardiomyopathy
DOX: Doxorubicin
ER: Endoplasmic reticulum
ERE: Estrogen response element
ERKs: Extracellular regulated protein kinases
GR: Glucocorticoid receptor
GRP78: Glucose regulated protein 78
GSK-3β: Glycogen synthase kinase-3β
H/R: Hypoxia/reoxygenation
HAECs: Human aorta endothelial cells
HCMECs: Human cardiac microvascular endothelial cells
HF: Heart failure
HO-1: Heme oxygenase-1
HPOA: Hypothalamus-pituitary–ovarian axis
HSF-1: Heat shock factor-1
HSPs: Heat shock proteins: Small heat shock proteins
HUVECs: Human umbilical vein endothelial cells
I/R: Ischemia/reperfusion
ICAM-1: Intercellular cell adhesion molecule-1
ICM: Ischemic cardiomyopathy
ISO: Isoproterenol
JNK: Jun N-terminal kinase
LAD: Left anterior descending
LPS: Lipopolysaccharide
MAPK: Mitogen-activated protein kinase
MCAO: Middle cerebral artery occlusion
MI: Myocardial infarction
mPTP: Mitochondrial MPT pore
NF-κB: Nuclear factor kappa-B
Nrf2: Nuclear factor E2-related factor2
NSCLC: Non-small cell lung carcinoma
NSCs: Neural stem cells
PARP: Poly (ADP-ribose) polymerase
PEDF-1: Serpin family F-1
PERK: dsRNA-activated protein kinase–like endoplasmic reticulum kinase
PI3K: Phosphatidylinositol 3-kinase
PPAR-γ: Peroxisome proliferators-activated receptor-γ
PRDX6: Peroxiredoxin 6
ROS: Reactive oxygen species
SCP-0-1: Homogeneous Schisandra chinensis polysaccharide-0-1
HSPs: Heat shock proteins: Small heat shock proteins
SIRT1: Silent information regulator1
TAC: Transverse aortic constriction
TCM: Traditional Chinese Medicine
TGF-β: Transforming growth factor-β
TLR4: Toll-like receptor 4
TNF-α: Tumor necrosis factor-α
UPR: Unfolded protein response
VEGF: Vascular endothelial growth factor
α-SMA: α-smooth muscle actin.
